# A Screening of Antineoplastic Drugs for Acute Myeloid Leukemia Reveals Contrasting Immunogenic Effects of Etoposide and Fludarabine

**DOI:** 10.3390/ijms21186802

**Published:** 2020-09-16

**Authors:** Darina Ocadlikova, Clara Iannarone, Anna Rita Redavid, Michele Cavo, Antonio Curti

**Affiliations:** 1 Dipartimento di Medicina Specialistica, Diagnostica e Sperimentale, Istituto di Ematologia “Seràgnoli”, Università degli Studi, 40138 Bologna, Italy; clara.iannarone@studio.unibo.it (C.I.); annarita.redavid@hotmail.it (A.R.R.); michele.cavo@unibo.it (M.C.); 2 Azienda Ospedaliero-Universitaria di Bologna, via Albertoni 15, 40138 Bologna, Italy; antonio.curti2@unibo.it

**Keywords:** acute myeloid leukemia, immunogenic cell death, chemotherapy, fludarabine, etoposide

## Abstract

Background: Recent evidence demonstrated that the treatment of acute myeloid leukemia (AML) cells with daunorubicin (DNR) but not cytarabine (Ara-C) results in immunogenic cell death (ICD). In the clinical setting, chemotherapy including anthracyclines and Ara-C remains a gold standard for AML treatment. In the last decade, etoposide (Eto) and fludarabine (Flu) have been added to the standard treatment for AML to potentiate its therapeutic effect and have been tested in many trials. Very little data are available about the ability of these drugs to induce ICD. Methods: AML cells were treated with all four drugs. Calreticulin and heat shock protein 70/90 translocation, non-histone chromatin-binding protein high mobility group box 1 and adenosine triphosphate release were evaluated. The treated cells were pulsed into dendritic cells (DCs) and used for in vitro immunological tests. Results: Flu and Ara-C had no capacity to induce ICD-related events. Interestingly, Eto was comparable to DNR in inducing all ICD events, resulting in DC maturation. Moreover, Flu was significantly more potent in inducing suppressive T regulatory cells compared to other drugs. Conclusions: Our results indicate a novel and until now poorly investigated feature of antineoplastic drugs commonly used for AML treatment, based on their different immunogenic potential.

## 1. Introduction

Recent evidence indicated that, under certain circumstances, chemotherapy stimulates the immune system. Indeed, in the last decade, some chemotherapeutic agents used for acute myeloid leukemia (AML) treatment, such as anthracyclines, have been shown to induce a type of cell death that can promote modifications in cancer cells, which activate the immune system against leukemia cells [1,2,3,4]. In particular, the treatment of AML cells with daunorubicin (DNR), but not cytarabine (Ara-C), results in the maturation of dendritic cells (DCs) and in the efficient cross-priming of anti-leukemia T cells [1,3,4,5,6,7]. This process, immunogenic cell death (ICD), is characterized by the coordinated emission of danger-associated molecular patterns (DAMPs), including the translocation of the endoplasmic reticulum (ER) chaperones such as calreticulin (CRT) and heat shock proteins (HSPs) 70 and 90 on the cell surface, the active secretion of adenosine triphosphate (ATP), the release of high mobility group box 1 (HMGB1) from the nucleus in the extracellular milieu [8,9,10,11,12] and, finally, the release of immunostimulatory cytokines, such as type I interferons [13,14]. In the early phase, CRT translocates from the ER to the outer leaflet of the plasma membrane, thus initiating the apoptotic caspase-dependent process [11,12]. Simultaneously, HSP70 and HSP90 bind tumor-associated antigens (TAAs), thus stimulating DC maturation [7,15]. During the late post-apoptotic phase, pro-inflammatory factor HMGB1—which binds the toll like receptor 4 (TLR4) on DCs—is released from the nucleus in the extracellular space [16]. Finally, autophagy-dependent active secretion of ATP—which binds purinergic receptors present on DCs—promotes the recruitment, survival and differentiation of DCs [17,18].

When emitted in the correct spatiotemporal context [12], these DAMPs recruit DCs in the proximity of ICD and activate them to engulf TAA processing and present them to CD4 and CD8 T cells, thus resulting in the priming of a robust, antigen-specific immune response [19,20].

Recent data support the role of chemotherapy in the activation of an anti-leukemia immune response [2,3,4,6,21], with important therapeutic implications. Indeed, Fredly et al. demonstrated that CRT is exposed by chemotherapy-treated primary human AML cells in 65% of tested patients. Moreover, in vitro cultured AML cells showed spontaneous release of HSP70 and 90 [6]. Recently, Fucikova et al. showed that CRT exposure by AML cells correlates with a strong anticancer immune response, improving the clinical outcomes of AML patients [3,22]. Prognostic statistical analysis of CRT, HSP70 and HSP 90 exposure revealed that ICD-associated DAMPs correlate with improved disease outcomes in patients with AML [3]. Wemeau et al. also demonstrated that CRT exposure on malignant blasts predicts a cellular anticancer immune response in patients with AML [4].

Although the scenario of AML is rapidly evolving and new targeted drugs are entering the clinical stage [23,24,25], the therapy of AML is at present principally based on the use of cytotoxic and cytostatic drugs, such as anthracyclines [26,27,28] and Ara-C [27,29,30]. Nevertheless, in induction chemotherapy, the conventional treatment regimen is able to induce complete remission (CR) in up to 70% of adult patients [31,32]. However, the probability of relapse remains elevated, particularly in elderly or prognostically “high risk” patients, unless transplantation of autologous or allogeneic hematopoietic stem cells is performed as a post-CR consolidation strategy [33]. To potentiate the effect of this drug combination, many attempts have been made in the last decades to add a third drug. In particular, Eto and Flu were tested in many clinical protocols [34,35,36]. Regarding the immunogenicity of these two drugs, too few studies have been reported in recent literature, and even fewer regarding AML [11,37,38,39,40]. We therefore studied the immunogenic potential of Eto and Flu as compared to DNR and Ara-C.

## 2. Results

### 2.1. DNR, Eto, Ara-C and Flu Induced a Similar Apoptosis Level in HL-60, KG-1 and Primary AML Cells

**Figure 1:**
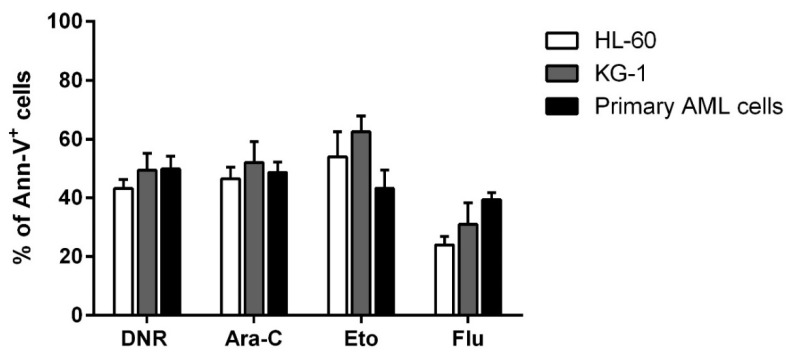
Flow cytometry analysis of acute myeloid leukemia (AML) cell apoptosis after chemotherapy treatment. The HL-60, KG-1 and primary AML cells were treated with daunorubicin (DNR) (500 ng/mL), cytarabine (Ara-C) (20 µg/mL), etoposide (Eto) (20 µg/mL) or fludarabine (Flu) (70 µg/mL) for 24 h. The percentage of apoptotic Ann-V^+^ cells was assessed by flow cytometry. The values are calculated as differences between treated and un-treated cells and represented as mean ± SEM of 5 independent experiments.

Before evaluating the immunogenic effect of tested chemotherapy drugs such as DNR, Eto, Ara-C and Flu, their cytotoxic activity on HL-60, KG-1 and primary AML cells was assessed to select the concentration of each drug capable of inducing similar and comparable levels of apoptosis. Various concentrations of each drug were tested. In particular, 10 µg/mL, 20 µg/mL, 50 µg/mL and 100 µg/mL for Eto, and 10 µg/mL, 20 µg/mL, 50 µg/mL, 70 µg/mL and 100 µg/mL for Flu were used (data not shown) to achieve an apoptosis level comparable to that previously determined for DNR and Ara-C [1]. As shown in Figure 1, the concentrations which induced a similar apoptosis level (percentage of annexin-V^+^ cells) to DNR and Ara-C treatment, were the following: 20 µg/mL for Eto with 53.9 ± 8.6%/62.4 ± 5.5%/43.2 ± 6.3% (HL-60/KG-1/primary AML cells, respectively), and 70 µg/mL for Flu with 24 ± 2.9%/31 ± 7.3%/39.4 ± 2.4%, of Annexin-V^+^ cells, respectively (mean ± SEM), as compared to 43.2 ± 3.1%/49.4 ± 5.7%/49.8 ± 4.3% for DNR at the concentration of 500 ng/mL, and 46.5 ± 39%/52 ± 7.1%/48.6 ± 3.6% for Ara-C at the concentration of 20 µg/mL.

### 2.2. Only Treatment with DNR and Eto, but Not Flu and Ara-C, Induced Translocation of CRT and HSPs from ER to Plasma Cell Membrane in AML Cells

**Figure 2:**
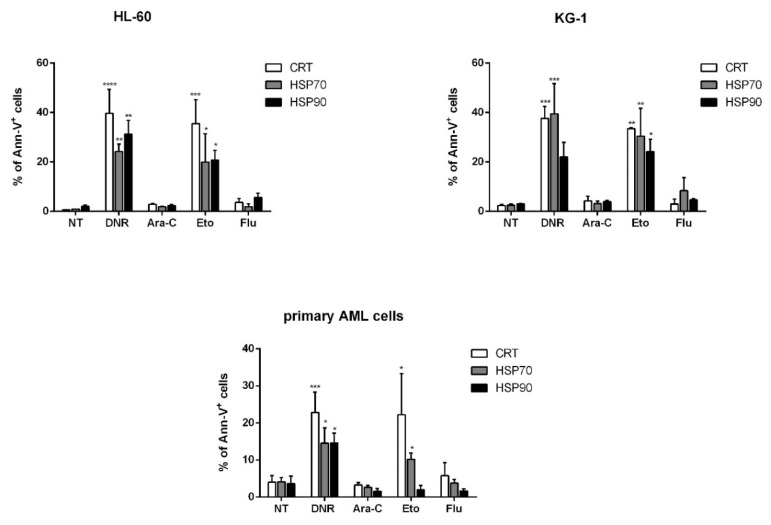
Flow cytometry analysis of calreticulin (CRT) and heat-shock protein (HSP) translocation on the cell-surface of acute myeloid leukemia (AML) cells after chemotherapy treatment. The HL-60, KG-1 and primary AML cells were treated with daunorubicin (DNR) (500 ng/mL), cytarabine (Ara-C) (20 µg/mL), etoposide (Eto) (20 µg/mL) and fludarabine (Flu) (70 µg/mL) for 24 h. The percentage of CRT^+^, HSP70^+^ and HSP90^+^ cells (gated on apoptotic Ann-V^+^ cells) was analyzed by flow cytometry. Un-treated cells (no treatment; NT) were used as a negative control. The values are represented as mean ± SEM of 5 independent experiments. * *p* <0.05; ** *p* < 0.01; *** *p* < 0.001; **** *p* < 0.0001 compared to un-treated cells.

ICD is represented by the coordinated emission of DAMPs, including CRT and HSPs 70 and 90 translocation from the ER to the cell surface, the active secretion of ATP and the active release of HMGB1 from the nucleus to the extracellular milieu [12]. We then in vitro tested and compared the capacity of each drug to induce these events. First, the evaluation of CRT, HSP70 and HSP90 exposure was determined in apoptotic cells by flow cytometry. HL-60, KG-1 or primary AML cells were treated with DNR (500 ng/mL), Ara-C (20 µg/mL), Eto (20 µg/mL) or Flu (70 µg/mL) for 24 h. The protein expression levels obtained by flow cytometry varied considerably among the different conditions (Figure 2). In particular, in the case of CRT, the expression significantly increased from 0.6 ± 0.1%/2.4 ± 0.4%/4 ± 1.8% (HL-60/KG-1/primary AML cells, respectively) in un-treated cells to 39.7 ± 9.7%/37.6 ± 4.9%/22.8 ± 5.5% in DNR-treated cells (*p* < 0.0001/0.001/0.001, respectively), or 35.5 ± 9.7%/33.5 ± 0.3%/22.2 ± 3.5% in Eto-treated cells (*p* < 0.001/0.01/0.05, respectively). In contrast, no significant differences were observed when Ara-C (2.9 ± 0.3%/4.3 ± 1.9%/3.3 ± 0.7%) or Flu (3.7 ± 1.5%/3 ± 2%/5.8 ± 3.5%) were used. Similarly, a significant HSP exposure was observed for HL-60, KG-1 and primary AML cells after DNR and Eto treatment, but not after Ara-C and Flu treatment (Figure 2). The only exception to this trend was represented by primary AML cells for HSP90 protein, where no up-regulation after Eto treatment was observed.

**Figure 3:**
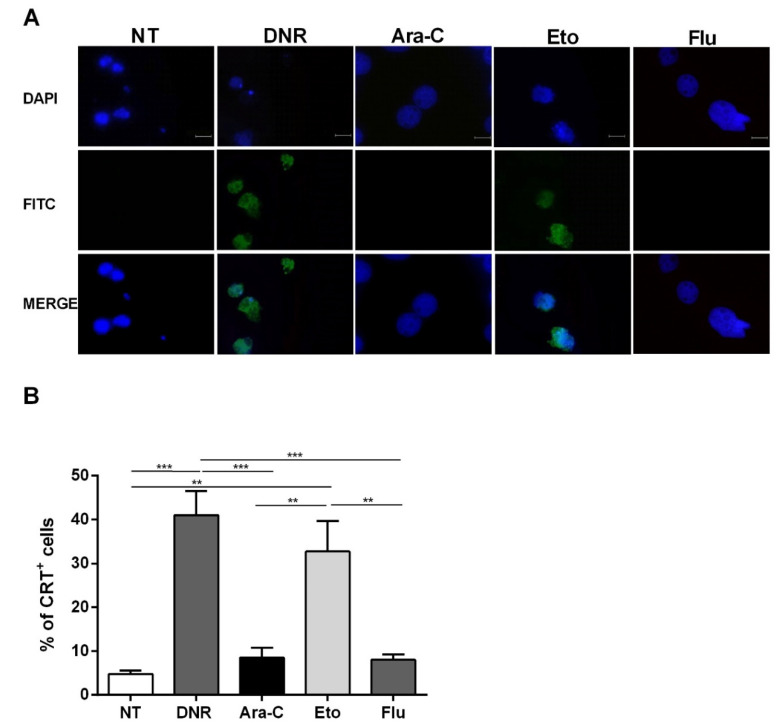
Immunofluorescence analysis of calreticulin (CRT) translocation on the cell-surface of HL-60 cell lines after chemotherapy treatment. The HL-60 cells were treated or not (no treatment; NT) with daunorubicin (DNR) (500 ng/mL), cytarabine (Ara-C) (20 µg/mL), etoposide (Eto) (20 µg/mL) and fludarabine (Flu) (70 µg/mL) for 24 h. (**A**) The localization of CRT (FITC-conjugated) at cell membrane level with respect to the nucleus (DAPI-conjugated) was evaluated by immunofluorescence microscopy. One representative experiment for each drug is reported. Bar 20 µm. (**B**) Quantitative analysis of CRT^+^ cells by immunofluorescence. A total of 100 cells were used for the quantification. ** *p* < 0.01; *** *p* < 0.001.

As shown in Figure 3, flow cytometry data regarding CRT translocation were also confirmed using immunofluorescence. Taken together, these in vitro data indicate that Eto is comparable to DNR in early ICD event induction. In contrast, Flu treatment is not capable of inducing either CRT or HSP translocation, similarly to Ara-C treatment.

### 2.3. Only Treatment with DNR and Eto, but Not Flu and Ara-C, Induced the HMGB1 Release from the Nucleus to the Extracellular Space of AML Cells

**Figure 4:**
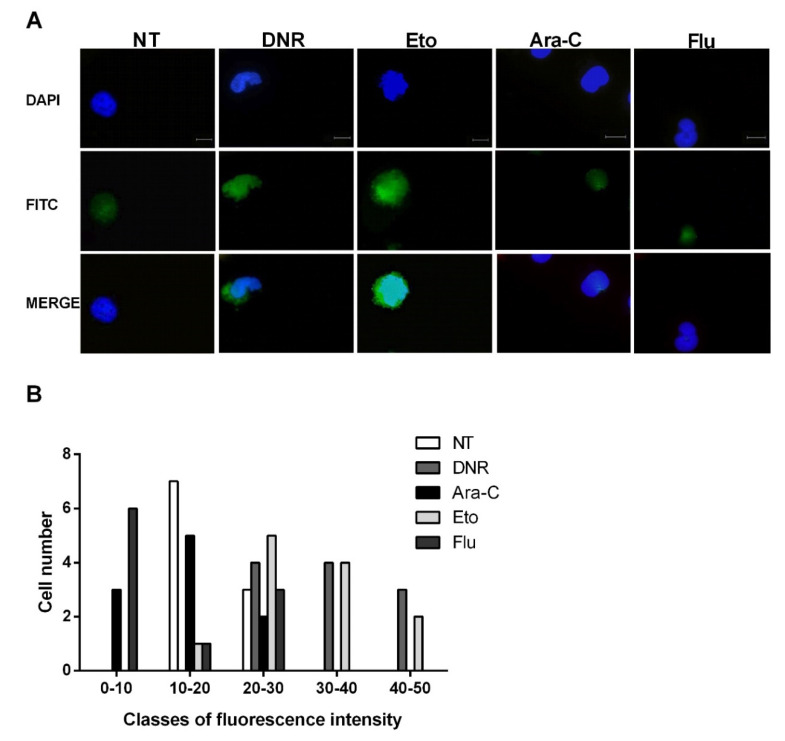
Immunofluorescence analysis of non-histone chromatin-binding protein high mobility group box 1 (HMGB1) release from the nucleus of HL-60 cell lines after chemotherapy treatment. The HL-60 cells were treated or not (no treatment; NT) with daunorubicin (DNR) (500 ng/mL), cytarabine (Ara-C) (20 µg/mL), etoposide (Eto) (20 µg/mL) and fludarabine (Flu) (70 µg/mL) for 24 h. (**A**) The release of HMGB1 (FITC-conjugated) from nucleus (DAPI-conjugated) to cytoplasm and then extracellular space was visualized by immunofluorescence microscopy. One representative experiment for each drug is reported. Bar 20 µm. (**B**) Quantitative analysis of HMGB1 fluorescence intensity outside the nucleus in un-treated and treated HL-60 cells. A representative field was used for quantification. The signal outside the nucleus was measured by densitometry (*n* = 21; randomly selected cells). The cells are grouped in classes of fluorescence intensity and plotted relative to HMGB1 expression.

After cellular stress, during the late post-apoptotic phase, pro-inflammatory nuclear factor HMGB1 translocates to the cytosol and is consequently released to the extracellular space [12]. After binding to specific receptors on DCs, HMGB1 induces the full maturation of DCs as evaluated by the up-regulation of CD40, CD54, CD80, CD83 and MHC II. To test this event, the HL-60 cells were treated with DNR (500 ng/mL), Ara-C (20 µg/mL), Eto (20 µg/mL) or Flu (70 µg/mL) for 24 h, then fixed, permeabilized, stained and analyzed for HMGB1 expression by immunofluorescence microscopy. As shown in Figure 4A, the extracellular release of HMGB1 was well-documented after DNR and Eto, but not after Ara-C and Flu, similarly to what was observed for CRT and HSP induction. These results were also confirmed when the HMGB1 expression density between un-treated and treated cells was evaluated (Figure 4B).

Collectively, in line with early ICD-related events, the immunofluorescence evaluation confirmed the presence of HMGB1 in the extracellular milieu after DNR (as expected) and Eto treatment, but not after Flu and Ara-C.

### 2.4. Only Treatment with DNR and Eto, but Not Flu and Ara-C, Induced ATP Release to the Extracellular Space of AML Cells

**Figure 5:**
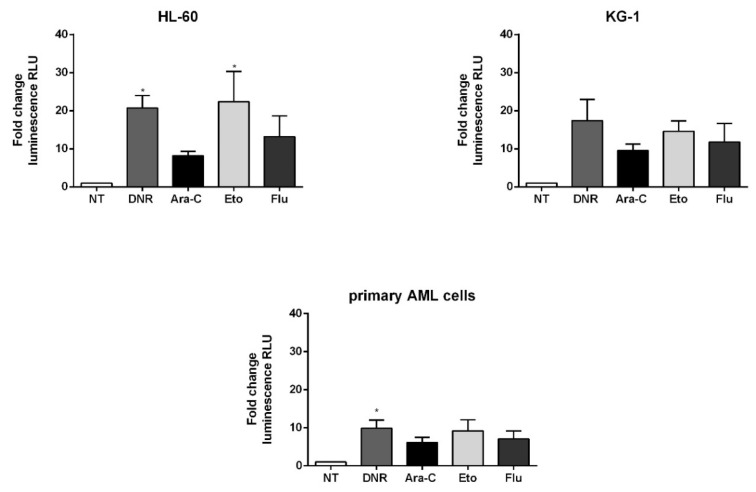
Luminescence analysis of adenosine triphosphate (ATP) release from HL-60, KG-1 and primary AML cells treated with chemotherapy. Indirect measurement of ATP by quantification of emitted bioluminescence in supernatants of HL-60, KG-1 and primary AML cells treated or not (no treatment; NT) with daunorubicin (DNR) (500 ng/mL), cytarabine (Ara-C) (20 µg/mL), etoposide (Eto) (20 µg/mL) and Fludarabine (Flu) (70 µg/mL) for 24 h, was expressed as fold change. The values of un-treated cells were equal to 1. The values are represented as mean ± SEM of 5/3/3 independent experiments for HL-60/KG-1/primary AML cells, respectively. * *p* < 0.05; compared to un-treated cells.

One of the most distinctive features of ICD is represented by the extracellular release of ATP from dying cells during the late apoptosis phase [17]. Autophagy-dependent active secretion of ATP, which binds purinergic receptors on DCs, promotes their recruitment, survival and differentiation [18]. ATP release was tested after 24 h by luminescence in HL-60, KG-1 or primary AML cells treated with DNR (500 ng/mL), Ara-C (20 µg/mL), Eto (20 µg/mL) or Flu (70 µg/mL) for 24 h. For HL-60 cells, comparable ATP levels were observed after DNR (fold change of luminescence 20.7 ± 5.8) and Eto treatment (22.4 ± 13.8), both significantly increased as compared to un-treated cells (*p* < 0.05), whereas both Ara-C and Flu treatment showed a lower capacity to induce ATP release (Figure 5).

A similar trend of higher ATP release after DNR and Eto treatment was observed also for KG-1 cells (Figure 5). Finally, a significant ATP release was observed after DNR treatment in primary AML cells (fold change of luminescence 9.9 ± 2.9) compared to un-treated cells (*p* < 0.05), whereas both Ara-C and Flu treatment showed a lower capacity to induce ATP release (Figure 5).

Collectively, these data indicate that, among the drugs that have been proposed to increase the efficacy of the conventional chemotherapy backbone including DNR and Ara-C, Eto has a similar and comparable capacity to DNR in inducing both early and late ICD events. On the contrary, Flu has a low if any effect, proving similar to Ara-C.

### 2.5. All Tested Drugs Induced DC Maturation Mediated by Chemotherapy-Treated HL-60, KG-1 and Primary AML Cells, but Only DNR and Eto Induced CD83 Up-Regulation, and Only DNR, Ara-C and Eto Induced CCR7 Expression

When emitted in the correct spatiotemporal context, the DAMPs recruit DCs in the proximity of ICD and activate them to engulf TAAs [12]. As a consequence, DCs become fully matured and competent in skewing cytokine production toward immunostimulation, a process which is strictly necessary for T cell priming and activation. The HL-60, KG-1 and primary AML cells were treated with DNR (500 ng/mL), Ara-C (20 µg/mL), Eto (20 µg/mL) or Flu (70 µg/mL) for 24 h and loaded in immature DCs (immDCs). After 24 h, the DC phenotype was evaluated by flow cytometry.

**Figure 6:**
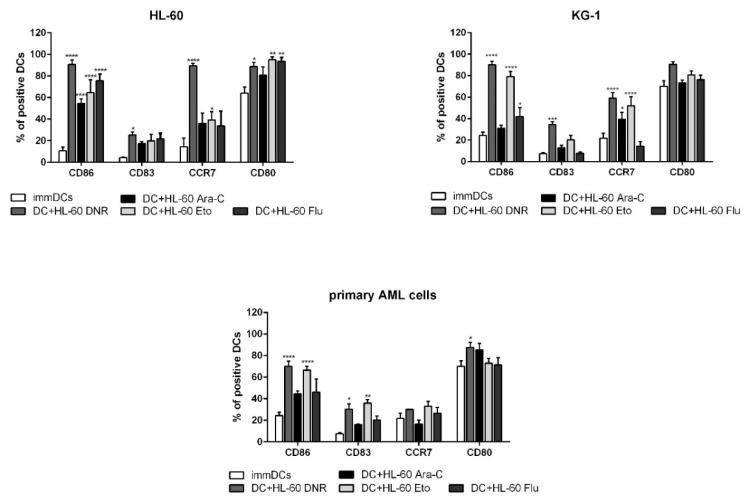
Flow cytometry analysis of dendritic cell (DC) maturation mediated by HL-60, KG-1 and primary AML cells treated with chemotherapy. The HL-60, KG-1 and primary AML cells were treated with daunorubicin (DNR) (500 ng/mL), cytarabine (Ara-C) (20 µg/mL), etoposide (Eto) (20 µg/mL) and fludarabine (Flu) (70 µg/mL) for 4 h and loaded in immature (imm) DCs for another 20 h. The DC phenotype was evaluated by flow cytometry. Un-loaded immature immDCs were used as a negative control. The values are represented as mean ± SEM of 5/3/3 independent experiments for HL-60/KG-1/primary AML cells, respectively. * *p* < 0.05; ** *p* < 0.01; *** *p* < 0.001; **** *p* < 0.0001 compared to immDCs.

As shown in Figure 6, all tested drugs induced a significant up-regulation of one or more DC-maturation markers, but only DNR significantly improved the expression of all of these (compared to immDCs), at least for HL-60 cells. For KG-1 and primary AML cells, DNR treatment significantly up-regulated three of four DC-maturation markers (Figure 6).

Very similar results were obtained after Eto treatment. In particular, a significant up-regulation of CD80, CD86 and CCR7, which is required for DC migration to lymph nodes, was observed in HL-60 cells. Interestingly, only DNR, Eto (in HL-60 and KG-1 cell lines) and also Ara-C (in KG-1 cell line) induced CCR7 expression among all tested cells (Figure 6).

Taken together, all four tested drugs induced a significant up-regulation of CD86 suggesting a partial DC maturation caused by the inflammatory microenvironment after chemotherapy treatment. Only DNR and Eto have the capacity to induce full DC maturation, including the up-regulation of CD83 and CCR7, which is known to regulate the capacity of DCs to migrate into T-cell enriched areas of draining lymph nodes.

### 2.6. Only DNR and Eto, but Not Flu and Ara-C, Induced the Proliferation of Allogeneic T Cells by DCs Loaded with HL-60, KG-1 and Primary AML Cells Treated Cells

DCs matured by loading with HL-60, KG-1 and primary AML cells treated cells become activated and may induce the proliferation of allogeneic T cells. DCs loaded for 24 h with HL-60, KG-1 and primary AML cells treated with DNR (500 ng/mL), Ara-C (20 µg/mL), Eto (20 µg/mL), Flu (70 µg/mL) or un-loaded immDCs were used in co-culture with allogeneic T cells. After 5 days, the T-cell proliferation by flow cytometry was evaluated.

**Figure 7:**
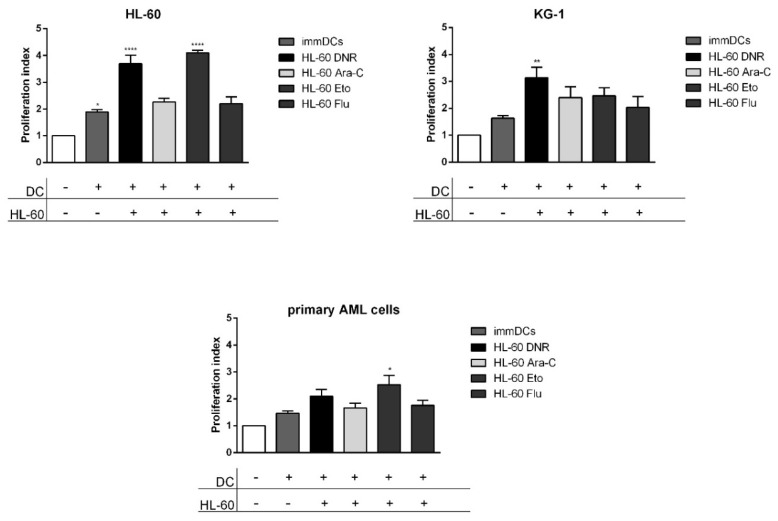
Flow cytometry analysis of CD3^+^ T-cell proliferation mediated by dendritic cells (DCs) loaded with chemotherapy-treated HL-60, KG-1 and primary AML cells. The HL-60, KG-1 and primary AML cells were treated with daunorubicin (DNR) (500 ng/mL), cytarabine (Ara-C) (20 µg/mL), etoposide (Eto) (20 µg/mL) and fludarabine (Flu) (70 µg/mL) for 4 h and loaded in immature DCs (immDCs) for 24 h. After 24 h, un-loaded immDCs or DCs loaded with treated HL-60, KG-1 and primary AML cells were used as a stimulus for CD3^+^ T cells for 5 days. The proliferation index of CD3^+^ T cells was then analyzed by flow cytometry and expressed as fold change. Un-stimulated CD3^+^ T cells were used as reference and set as 1. The values are represented as mean ± SEM of 5/3/3 independent experiments for HL-60/KG-1/primary AML cells, respectively. * *p* < 0.05; ** *p* < 0.01; **** *p* < 0.0001 compared to un-stimulated CD3^+^ T cells.

For HL-60 cells, the autologous un-loaded immDCs induced a modest proliferation (proliferation index 1.89) compared to un-stimulated CD3 (1), as shown in Figure 7. The proliferation status improved significantly after adding DCs, previously loaded with HL-60 treated with DNR or Eto, as compared to un-stimulated CD3. In particular, Eto induced the highest proliferation index (4.1) (Figure 7). On the contrary, Flu treatment was capable of inducing little increase of T-cell proliferation (2.2) over un-loaded immDCs (1.89).

A significant up-regulation of proliferation index was observed also after DNR treatment for KG-1 cells (3.1) and after Eto treatment for primary AML cells (2.5) compared to un-stimulated CD3 (1), as shown in Figure 7.

Collectively, our data demonstrate that Eto can be considered an ICD inducer comparable to DNR.

In particular, along with the induction of all ICD-related events and full DC maturation, Eto treatment was the most powerful among the tested drugs in stimulating T-cell proliferation, thus suggesting a significant capacity to activate the immune response. On the contrary, Flu had weak immunogenic potential and can be considered a non-immunogenic chemotherapy drug.

### 2.7. Flu-Treated Leukemic Cells Induced a Population of Suppressive T Regulatory Cells via DCs

To test the tolerogenic potential of the drugs, the induction of T regulatory cells (Tregs) was evaluated. DCs loaded for 24 h with HL-60, KG-1 and primary AML cells treated with DNR (500 ng/mL), Ara-C (20 µg/mL), Eto (20 µg/mL), Flu (70 µg/mL), or un-loaded immDCs were used in co-culture with allogeneic T cells. After 5 days, the total Tregs induction, characterized by the expression of CD3^+^CD4^+^CD25^+/high^CD127^−/low^, as well as the suppressive Tregs subpopulation characterized by the expression of CD3^+^CD4^+^CD25^high^CD127^−/low^CD45RA^−^FOXP3^+/high^, was evaluated by flow cytometry.

**Figure 8:**
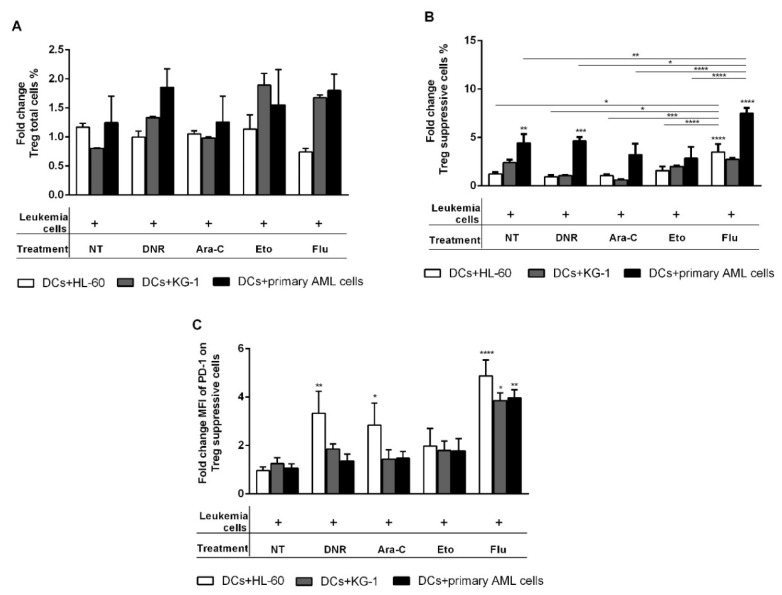
Flow cytometry analysis of T regulatory cells (Tregs) induced by dendritic cells (DCs) loaded with chemotherapy-treated HL-60, KG-1 and primary AML cells. The HL-60, KG-1 and primary AML cells were treated or not (no treatment; NT) with daunorubicin (DNR) (500 ng/mL), cytarabine (Ara-C) (20 µg/mL), etoposide (Eto) (20 µg/mL) and fludarabine (Flu) (70 µg/mL) for 4 h and loaded in immature DCs (immDCs) for 24 h. After 24 h, un-loaded immDCs or DCs loaded with treated HL-60, KG-1 and primary AML cells were mixed with CD3^+^ T cells for 5 days. The Tregs phenotype was then analyzed by flow cytometry. (**A**) Induction of total Tregs was characterized as CD3^+^CD4^+^CD25^+/high^CD127^−/low^ T cells and expressed as fold change. (**B**) Induction of suppressive subtype of Tregs was characterized as CD3^+^CD4^+^CD25^high^CD127^−/low^ CD45RA^−^/FOXP3^+/high^ T cells and expressed as fold change. (**C**) Mean of fluorescence intensity (MFI) of programmed cell death protein 1 (PD-1) expressed on suppressive Tregs was expressed as fold change. The values are represented as mean ± SEM of 5/3/3 independent experiments for HL-60/KG-1/primary AML cells, respectively. CD3 cells stimulated with unloaded immDCs were used as reference and set as 1. * *p* < 0.05; ** *p* < 0.01; *** *p* < 0.001; **** *p* < 0.0001 compared to CD3 cells stimulated with un-loaded immDCs.

As shown in Figure 8A, none of the tested drugs significantly induced a total population of Tregs, but, interestingly, Flu induced a significant number of suppressive Tregs (Figure 8B), as compared to other drugs. In particular, DCs loaded with HL-60 cells treated with Flu induced 3.5 ± 0.8 (fold change) of suppressive Tregs compared to un-loaded DCs (*p* < 0.0001) or DCs loaded with DNR-/Ara-C/Eto-treated HL-60 cells (0.9 ± 0.2/1.1 ± 0.1/1.6 ± 0.4, respectively; *p* < 0.05/*p* < 0.001/*p* < 0.0001). A similar pattern was observed when DCs loaded with primary AML cells treated with Flu (fold change 7.5 ± 0.5) were used for Tregs induction compared to un-loaded DCs (*p* < 0.0001) (Figure 8B). Similarly to HL-60 cells, DCs loaded with primary cells treated with Flu also induced a significant up-regulation of suppressive Tregs compared to DNR/Ara-C/Eto (*p* < 0.05/*p* < 0.0001/*p* < 0.0001, respectively). For DCs loaded with Flu-treated KG-1 cells, only a trend in suppressive Tregs up-regulation was observed (fold change 2.7 ± 0.1). Moreover, a more in-depth characterization of Flu-induced suppressive Tregs revealed an up-regulation of programmed cell death protein 1 (PD-1) expression indicating their potentiality and effector function (Figure 8C). The fold change of the mean of fluorescence intensity (MFI) of PD-1 expressed on suppressive Tregs increased to 4.9 ± 0.7, when DCs loaded with Flu-treated HL-60 were used (*p* < 0.0001 compared to unloaded DCs). Similarly, DCs loaded with KG-1 or primary AML cells also induced a significant up-regulation of PD-1 MFI on suppressive Tregs to 3.9 ± 0.3 or 4.0 ± 0.3, respectively (fold change), after Flu treatment compared to un-loaded DCs (*p* < 0.05 or *p* < 0.01, respectively) (Figure 8C). Interestingly, we observed an up-regulation of PD-1 also after DNR and Ara-C, but not Eto treatment, when DCs loaded with HL-60 cells were used to induce Tregs. These data are in line with previously obtained results highlighting the contrasting immunological effect of Flu and Eto treatment.

Taken together, these data indicate Flu as a non-immunogenic chemotherapy drug with suppressive effects on the immune system and are in line with previous results obtained in this study.

## 3. Discussion

Our results demonstrate that, among the four anti-leukemia drugs commonly used in AML treatment, Eto is comparable to DNR in ICD-related event induction, whereas Flu, similarly to Ara-C, has a weak immunogenic effect and, interestingly, may increase Tregs.

In recent years, a growing body of evidence has shed new light on the composition of the immunological microenvironment in AML patients. Multiple and contrasting aspects of T cell function are operative in AML bone marrow (BM) at diagnosis, such as activation along with exhaustion and senescence. These data reveal the capacity of AML, similarly to solid tumors, to shape and edit anti-leukemia immune response. Although in solid tumors the effect of some chemotherapy and targeted agents on the tumor immunological microenvironment is well-established [41], the impact of chemotherapy on immune response in AML has not been extensively investigated. Recent evidence indicates the elasticity of AML cells in modulating CD8^+^ T cell responses and the plasticity of their signatures upon chemotherapy response, which is capable of reversing some dysfunctional features of BM-infiltrating T cells [42]. These data extend to the AML field the notion that chemotherapy drugs are important not only to directly eliminate tumor cells but also to induce or reinforce immune system responses that may be crucial for the eradication of chemo-resistant malignant cells [43]. In this scenario, our work expands our knowledge regarding the immunogenic potential of the chemotherapy drugs that are commonly used as an induction regimen in AML patients. We and others have previously demonstrated that DNR is a very strong ICD inducer [1,3,44], whereas Ara-C has a weak immunogenic capacity. Interestingly, Eto and Flu, which are alternatively combined with the conventional DNR and Ara-C-based induction regimen with the aim of increasing its effectiveness, proved to be profoundly different in their immunogenic activity. Very similarly to DNR, Eto is a strong ICD inducer. Recently, it was shown that Eto induces cell apoptosis through a mechanism involving the ER stress pathway [45]. In the ICD context, it was well demonstrated that phosphorylation of eukaryotic initiation factor 2 (eIF2α) is essential for the ER stress response and is correlated with CRT/ERp57 complex exposure, leading to DC activation in various tumor models [46,47,48,49]. These findings support our hypothesis that a molecular target of Eto inducing ICD process could be the eIF2α inducing the ER stress. Moreover, Cheng et al. demonstrated that Eto in combination with 2-deoxyglucose (2-DG; inhibitor of glycolysis), but not 2-DG alone, induces ICD in mouse lymphoma model, and that this effect was at least partially mediated through CRT exposure on the plasma membrane. This is a first sign that Eto also has immunogenic properties in other tumor model [45,50].

Contrary to DNR and Eto, Flu, more similarly to Ara-C, reduced ICD capacity and, interestingly, may directly exert a tolerogenic function by inducing a population of suppressive Tregs. These data are intriguing and could be correlated with the recent evidence that different immunological landscapes exist in cancer, including AML, that are associated with important and clinically relevant differences in chemosensitivity as well as in response to immunotherapy approaches [51].

In AML, inflammatory patterns as well as inhibitory signals, such as the expression of immune checkpoint receptors on leukemic cells [52], have been recently associated with high-risk cytogenetic and molecular profile, which in turn associates with resistance to standard chemotherapy, including anthracyclines [31,53]. In this scenario, we may speculate that the use of proinflammatory chemotherapy drugs, such as Eto and DNR, may further increase a condition of T-cell exhaustion, correlated with inflammation. In contrast, anti-inflammatory, even tolerogenic, drugs, such as Flu, may favor the induction of anti-tumor immunity by preventing T-cell anergy and exhaustion. Indeed, a better understanding of the immunologic effects of chemotherapy drugs, along with the use of immunogenomics in the in-depth characterization of the AML microenvironment, may guide the clinical choice toward a more personalized and immunological-driven use of chemotherapy and targeted agents in AML.

It is well-known that the AML microenvironment is mostly enriched in Tregs, which interacts with effector T cells, thus crucially dampening the anti-leukemia immune response and favoring leukemia immunological escape [54,55]. In particular, the role of Tregs in AML is very important for both the characterization of the BM microenvironment composition before chemotherapy and the prediction of response to chemotherapy [56,57,58,59]. Indeed, as compared to healthy individuals, AML patients at diagnosis may have higher numbers of Tregs, whose frequency is directly correlated with response to chemotherapy [57,60], and a rapid turnover of Tregs after chemotherapy has been demonstrated in AML patients [58]. Our group has recently addressed the mechanisms by which DNR and Ara-C may contribute to modify the immune response in AML patients and in a mouse model of AML [1]. Briefly, we demonstrated that especially DNR, along with the activation of anti-leukemia immunity, may also induce tolerance by increasing the number of leukemia-infiltrating tolerogenic DCs and, more importantly, by expanding a population of Tregs. These findings suggested that Tregs induced after DNR treatment may play an important regulatory role in the choice between tolerance and immunity in response to chemotherapy-treated dying leukemia cells and are in line with other recent studies which use preclinical models of self-tolerance and autoimmunity [61]. The in vitro results of the present study extend our knowledge on the tolerogenic capacity of other drugs commonly used in the therapy of AML, such as Eto and Flu. In particular, Flu proved to be a potent inducer of Tregs when used to treat AML cells before in vitro cultures, whereas Eto has a weak capacity to induce Tregs, while maintaining T-cell stimulatory function. These results are not surprising, given the well-established immunosuppressive activity of Flu, especially in the context of lymphomas and chronic lymphocytic leukemia [62]. However, to our knowledge, this is the first demonstration that, when used to pulse DCs in T-cell cultures (cross-priming effect), Flu-treated AML cells may potently act as a Treg inducer, thus providing a new immunosuppressive mechanism associated with Flu administration. Moreover, our data indicate that Tregs obtained after cultures with DCs pulsed with Flu-treated AML cells have the highest expression of PD-1 among all tested drugs. It is known that PD-1 could be highly expressed on Tregs and is fundamental for the inhibition of effector T cell function as well as for the induction and maintenance of T cell tolerance via Tregs [63,64]. Accordingly, the expression of PD-1 on Tregs correlates with their immunosuppressive activity [65,66,67], and the accumulation of PD1^+^Foxp3^+^ Tregs within the tumor microenvironment of solid tumors strongly supports their immunosuppressive potential [65,67]. In AML patients, PD-1 expression was observed in different T-cell subpopulations, including Tregs [52]. Our group recently confirmed these data in AML, showing higher PD-1 expression on Tregs in both in vivo mouse models and AML patients after chemotherapy treatment [1]. Since the PD-1/PD-1 ligand axis represents a target of therapy in many clinical studies for solid tumors and leukemias including AML [44], the up-regulation of PD-1 on Tregs after AML treatment with Flu may have interesting clinical implications. In particular, the use of anti-PD-1 checkpoint inhibitors in combination with chemotherapy has the potential of targeting Tregs, which prominently contributes to the tolerogenic microenvironment in AML.

Taken together and with the limitations of an in vitro study, the present investigation expands the knowledge on the immunogenic and tolerogenic potential of the chemotherapy drugs commonly used in the therapy of AML. Among these, important differences have been observed, indicating that, particularly in an era when immunotherapy is being included in the clinical stage of AML treatment, the immunological perspective of chemotherapy should be taken into consideration in therapy decision-making. As a future goal, a set of in vivo studies are planned to confirm these in vitro data.

## 4. Materials and Methods

### 4.1. Cells

Human HL-60 (ACC 3, FAB M2) and KG-1 (ACC14, relapse) AML cell lines were obtained from DSMZ (Leibnitz Institute, German Collection of Microorganisms and Cell Cultures; Leibnitz; Germany). Primary AML cells were obtained as mononuclear cells separated by Ficoll–Hypaque centrifugation (Amersham, Buckinghamshire, UK) from 3 newly diagnosed AML patients (1. 46 XX, M1; 2. 46 XY, M2; 46 XY, del9(q21;q34), M2; karyotype and Fab, respectively) after informed consent signature (local ethics committee approval code: 147/2013/O/Tess; approved 11 June 2013). The cells were cultured in RPMI 1640 medium (Lonza, Milan, Italy), supplemented with 10% heat-inactivated fetal bovine serum (FBS; Sigma Aldrich, St. Louis, MO, USA), 2 mM L-glutamine, 100 U/mL penicillin and 100 µg/mL streptomycin (MP Biomedicals, Milano, Italy) (complete RPMI) and maintained at 37 °C and 5% CO_2_. CD3^+^ and CD14^+^ cells were purified by magnetic separation (MiltenyiBiotec, Bergisch Gladbach, Germany), according to the manufacturer’s instructions from mononuclear cells separated from buffy coats of healthy donors by Ficoll–Hypaque centrifugation (Amersham; Burlington; Canada) after informed consent signature (local ethics committee approval code 94/2016/O/TES). Purity of cell populations was always >90%.

### 4.2. HL-60, KG-1 and Primary AML Cell Treatment

HL-60, KG-1 and primary AML cells were treated with DNR 500 ng/mL (Sigma-Aldrich, St. Louis, MO, USA), Ara-C 20 µg/mL (Sigma-Aldrich), Flu 70 µg/mL (Sigma-Aldrich) or Eto 20 µg/mL (Sigma-Aldrich) for 4 h, washed and used for DC pulsing or reseeded for another 20 h and then tested for apoptosis by Annexin-V-FLUOS Apoptosis Detection Kit (Roche, Basel, Switzerland), according to the manufacturer’s instructions, and flow cytometry staining (see below). ATP release and immunofluorescence staining (see Section 4.4 and Section 4.5, respectively) were performed only with HL-60 cell lines.

### 4.3. CRT and HSP70 and 90 Staining by Flow Cytometry

One hundred thousand HL-60, KG-1 or primary AML cells treated with DNR, Ara-C, Flu and Eto as described above or un-treated were stained with human-specific primary monoclonal antibodies (mAbs) for CRT (AB92516; Abcam, Cambridge, UK), HSP70 (AB181606; Abcam) and HSP90 (AB13495; Abcam) at dilution ratios of 1:100, 1:230 and 1:250, respectively, in blocking solution (PBS/FBS 2%). After 30 min of incubation, the cells were washed with cold PBS and stained for another 30 min in the dark with secondary mAb Donkey Anti-Rabbit IgG-AlexaFluor 647 (AB150075, Abcam) diluted at 1:5000. After a final wash with cold PBS, the cells were stained with Ann-V by Annexin-V-FLUOS Apoptosis Detection Kit (Roche) for 15 min and then analyzed on flow cytometer BDAccuriC6 (BD Biosciences, Franklin Lakes, WI, USA). At least 10,000 events were analyzed. The cells stained only with secondary mAb for each condition were used as negative fluorescence control.

### 4.4. Quantification of ATP Release

HL-60, KG-1 or primary AML cells were seeded in 96-well flat bottom plates (1 × 10^6^/mL) and treated with chemotherapeutic agents DNR, Ara-C, Flu and Eto (as described above), for 4 h. Cells were then washed and after 20 h ATP quantification in the supernatants was performed in triplicate using ENLITEN rLuciferase/Luciferin Reagent (Promega, Madison, WI, USA), according to the manufacturer’s instructions. Luminescence was measured at the single-tube luminometer Glomax 20/20 (Promega), with 10-second RLU (relative light units) signal integration time.

### 4.5. CRT and HMGB1 Staining by Immunofluorescence

HL-60, KG-1 or primary AML cells (500,000/condition) treated with DNR, Ara-C, Flu and Eto, as described above, or un-treated were re-suspended in PBS and centrifuged by Cytospin (Shandon-Elliott Instruments Limited, Runcorn, UK) for 10 min at 1000 rpm, speed 40. The samples were then fixed with 4% paraformaldehyde for 10 min. After repeated washing with cold PBS, the cells were stained according to the following protocols. The images were acquired and processed by Axiovert 40 CFL microscope (Carl Zeiss Microscopy-LLC, New York, NY, USA).

#### 4.5.1. CRT Exposure

First, the cells were treated with blocking solution (PBS + 5% bovine serum albumin; BSA from Sigma-Aldrich) for 30 min. Then, primary antibody anti-human calreticulin (AMAB29516; Abcam) diluted at 1:100 in blocking solution was used for staining. After 30 min of incubation, the cells were washed with cold PBS and stained with secondary antibody Alexa Fluor 488 anti-rabbit (A27034; Life technologies/ThermoFisher Scientific, Waltham, MA, USA) at a concentration of 5 µg/mL in blocking solution for 30 min in the dark. After the last wash in cold PBS, one drop of ProLong Gold Antifade Mountant reagent with DAPI (Thermofisher) was added. The slide was closed with transparent nail polish and stored at −20 °C in the dark before microscope analysis. For quantitative analysis of CRT^+^ cells by immunofluorescence, a total of 100 cells were used for quantification.

#### 4.5.2. HMGB1 Release

First, the cells were washed with 0.1% PBS-Tween (Sigma Aldrich) and permeabilized with 0.2% PBS-Triton (Sigma-Aldrich) for 10 min. After another washing with 0.1% of PBS-Tween, the cells were incubated for 30 min in blocking solution (PBS + 5% of BSA) and stained with primary antibody anti-human HMGB1 (3935S; Cells Signaling, Danvers, MA, USA) diluted at 1:100 in blocking solution for 30 min. The cells were then washed with 0.1% PBS–Tween and stained with secondary antibody Alexa Fluor 488 anti-rabbit (A27034; Life technologies/ThermoFisher Scientific) at a concentration of 5 µg/mL in blocking solution for 30 min in the dark. After the last wash in 0.1% PBS–Tween, one drop of ProLong Gold Antifade Mountant reagent with DAPI (Thermofisher) was added. The slide was closed with transparent nail polish and stored at −20 °C in the dark before microscope analysis.

The immunofluorescence intensity was measured by densitometry using Photoshop (Adobe Photoshop software 6.0). The cells were grouped in classes of low, intermediate and high fluorescence intensity (6 cells per group). The values were corrected by pixel number to compare cells with different dimensions [68].

### 4.6. DC Generation, Pulsing and Maturation

Human monocyte-derived DCs were generated by a 5-day culture of CD14^+^ cells in complete RPMI in the presence of granulocyte-macrophage colony-stimulation factor (50 ng/mL; GM-CSF Endogen, Worldwide, St. Louis, MO, USA) and IL-4 (800 U/mL; MiltenyiBiotec), as previously described [69,70]. DC maturation was induced by pulsing with treated or un-treated HL-60, KG-1 or primary AML cells, as described in Section 4.2. For DC pulsing, chemotherapy-treated HL-60, KG-1 or primary AML cells were cultured for 20 h with immDCs (2:1 ratio) in complete RPMI. After culture, mature and immDCs were tested for immunophenotype and used for proliferation and Treg induction testing.

### 4.7. DC Phenotype by Flow Cytometry

Mature and immDCs were stained for 15 min in the dark using the following anti-human mAbs: HLA-DR FITC (clone L243; BD Biosciences), CD14 PE (clone 61D3; eBioscience/ThermoFisher, Waltham, MA, USA), CD86 PECy7 (clone IT2.2; eBioscience/ThermoFisher), CD83 PE (clone HB15; Biolegend), CD80 APC (clone 2D10; Biolegend, San Diego, CA, USA) and CCR7 Alexa Fluor 647 (clone G043H7; Biolegend). For each sample, unstained DCs were used as negative fluorescence control. At least 10,000 events of each sample were collected and analyzed using a FACS Canto II Flow Cytometer (BD Biosciences).

### 4.8. Proliferation Test

Twenty thousand immDCs, chemotherapy-treated HL-60, KG-1 or primary AML cells pulsed DCs (as described in Section 4.6) were irradiated at 3000 cGy and co-cultured for 5 days in complete RPMI with 200,000 autologous CD3^+^ T cells at the ratio of 1:10. The CD3^+^ T cells were stained before the co-culture with 5 µM of carboxyfluorescein succinimidyl ester (CFSE; Abcam). The proliferation was analyzed on a FACS Canto II (BD Biosciences) flow cytometer, and the proliferation index was calculated using FCS express 6 software.

### 4.9. Treg Induction

Twenty thousand immDCs, chemotherapy-treated HL-60, KG-1 or primary AML cells pulsed DCs (as described in Section 4.6) were co-cultured for 5 days in complete RPMI with 200,000 autologous CD3^+^ T cells at the ratio of 1:10. The CD3^+^ T cells alone were used as negative control. After 5 days of co-culture, T cells were stained for 15 min in the dark using the following anti-human mAbs: CD4 APCH7 (clone SK3; BD Biosciences), CD25 PeCy7 (clone BC96; Biolegend), CD127 PerCP 5.5 (clone A019D5; Biolegend), CD45RA V500 (clone HI100; Biolegend), PD-1 APC (clone EH12.2H7; Biolegend). Intracellular staining of FOXP3 using Foxp3/Transcription Factor Staining Buffer Set (eBioscience/ThermoFisher) was performed as follows. For each sample, unstained CD3 cells were used as negative fluorescence control. At least 5000 events of Total Tregs in each sample were collected and analyzed using a FACS Canto II Flow Cytometer (BD Biosciences).

## Abbreviations

AML	Acute myeloid leukemia
DNR	Daunorubicin
Ara-C	Cytarabine
ICD	Immunogenic cell death
Eto	Etoposide
Flu	Fludarabine
DCs	Dendritic cells
DAMPs	Danger associated molecular patterns
ER	Endoplasmic reticulum
CRT	Calreticulin
HSPs	Heat shock proteins
ATP	Adenosine triphosphate
HMGB1	Non-histone chromatin-binding protein high mobility group box 1
TAAs	Tumor associated antigens
TLR4	Toll like receptor 4
CR	Complete remission
NT	No treatment
immDCs	Immature DCs
Tregs	T regulatory cells
PD-1	Programmed cell death protein 1
BM	Bone marrow
mAbs	Monoclonal antibodies
MFI	Mean of fluorescence intensity
2-DG	2-Deoxyglucose
